# The Ethnobotany and Chemistry of South African Meliaceae: A Review

**DOI:** 10.3390/plants10091796

**Published:** 2021-08-28

**Authors:** Mariam Oyefunke Oyedeji-Amusa, Nicholas J. Sadgrove, Ben-Erik Van Wyk

**Affiliations:** 1 Department of Botany and Plant Biotechnology, University of Johannesburg, P.O. Box 524, Auckland Park 2006, South Africa; oyedejiamusa@gmail.com (M.O.O.-A.); n.sadgrove@kew.org (N.J.S.); 2 Jodrell Science Laboratory, Royal Botanic Gardens, Kew, Richmond TW9 3DS, Surrey, UK

**Keywords:** South African Meliaceae, ethnomedicinal importance, functional uses, chemistry, limonoids

## Abstract

Meliaceae are widely distributed across the world in tropical or subtropical climates and are of considerable ethnobotanical importance as sources of traditional medicine and cosmetics. This comprehensive review summarizes the ethnobotanical uses and chemistry of 12 South African species, belonging to six genera: *Ekebergia, Nymania, Entandrophragma, Pseudobersama, Trichilia,* and *Turraea*. Eight of the species have ethnomedicinal records, classified into 17 major disease categories. The ethnomedicinal uses comprise 85 ailments dominated by gastrointestinal complaints, followed by gynaecological and obstetrics related problems. Chemical records were found for 10 species, which describe nine classes of compounds. In nearly all South African Meliaceae, limonoids are the predominant constituents while triterpenes, sterols, and coumarins are also common. The widest range of use-records and medicinal applications are found with the two most chemically diverse species, *Ekebergia* *capensis* and *Trichilia* *emetica*. Of the chemical compounds identified in the various plant organs of the 10 species of South African Meliaceae for which data are available, 42% was found in bark and 17% in seeds. Roots represent 35% and bark 33% of the organs that are used medicinally, and they are typically prepared as decoctions or infusions. Root and bark harvesting are destructive so that it may be important to examine the chemistry of plant parts such as wild-crafted leaves and fruits.

## 1. Introduction

Ethnobotany is the cultural study of the practical uses of a region’s plants by the local people. It is interdisciplinary and can often progress into a lab-based collaborative project with the vision of benefiting modern society in the form of wild food crops, pharmaceuticals, nutraceuticals, or cosmetics [1]. Furthermore, by recording traditional plant use, indigenous knowledge and belief systems are conserved [2] and incentives for biodiversity conservation are realized. Unfortunately, the floras that are used traditionally are the most likely to be destroyed or threatened by over-exploitation. The management of plants that are utilised by local people or small grass-roots level industries may be facilitated by a more complete understanding of the dynamics of people–plant interactions [2,3,4].

The culture of plant-based subsistence is rapidly becoming a rarity in the modern world. Hence, the cultures of the African people represent a minority that have continued practicing holistic environmentalism that utilises raw plant-based materials for food, medicines, as pesticides or tools and in spiritual pursuits including rituals [5]. In the modern day, most South Africans rely on traditional medicine as a first line of treatment. This is chiefly due to its affordability, accessibility, and the high level of knowledge by local traditional healers [6,7]. In this regard, about 3000 out of over 20,000 species of higher plants in South Africa are used in traditional medicine [8]. The botanical prescriptions made by the archetypical traditional healers in South Africa are collectively called ‘muthi’ and are generally distributed out of informal markets.

Since the turn of the 21st century, there has been a renewal of interest in the use of medicinal plants and herbal remedies for the treatment of health afflictions or in nutritional support [9,10]. While traditional healing systems in Africa have become a rich source of information on plant-based health, there are minimal written records to draw upon to guide the integration of natural products into developed societies. This is due to the fact that medicinal plant-use knowledge of traditional healers is passed on from generation to generation via word of mouth [6]. Hence, the importance of creating written records of traditional knowledge cannot be over emphasized, particularly in South Africa [2] or in highly remote locations such as Ethiopia [5]. Pappe [11] published the first synthesis of South African medicinal plants, then nearly a century later, Watt and Breyer-Brandwijk [12] provided a more comprehensive report on medicinal plants used in the same country. Hutchings et al. [13] created a focused account of medicinal plants commonly used in the Zulu nation, which was further elaborated by Van Wyk et al. [6] and especially by Mhlongo and Van Wyk [14]. 

The chemistry of taxa from South African Meliaceae is highly diverse. Many sesquiterpenes, sterols, coumarins, flavonoids, and other phenolics have been reported. Species in Meliaceae are well known for their bitter and biologically active nortriterpenoids, known as limonoids or meliacins [15,16]. Over 300 limonoids have been isolated from the world’s flora and their production is confined to the order Rutales, of which they are more diverse and abundant in Meliaceae than in any other family [15,16,17]. They are derivatives of 4,4,8-trimethyl-17-furanylsteroid. These compounds have aroused considerable commercial interest due to their molluscicidal, antifungal, bactericidal, insect-antifeedant, insect-repellent, insecticidal, and plant antiviral activities, as well as their numerous medicinal effects in humans and animals [16,18,19,20]. Hence, limonoids have attracted significant interest within biological and chemical research disciplines.

Several researchers have reported the chemistry, biosynthesis, and biological activities of meliaceous limonoids [21,22,23,24,25]. *Azadirachta indica* L. is known as a famous limonoid producing plant as well as a source of environmentally friendly biopesticide of commercial importance in the agricultural sector. Products of *A. indica* (such as align, azitin, margosan-O, and turplex) were recognized and approved as pest control agents in the United States [26]. In China, three commercial limonoid products (from *A. indica*, *Melia azedarach* L., and *Melia toosendan* Siebold & Zucc.) were granted approval for insect controls on organic vegetable plantings.

The ethnobotanical uses of taxa in South African Meliaceae are well documented [6,12,13,27,28,29,30]. Several limonoids and other secondary metabolites with appreciable biological activities have also been reported in South African species of Meliaceae [31,32,33,34,35]. Hence, the aim of this study is to present a detailed and comprehensive review of the ethnobotanical uses and compounds that have been previously isolated from South African indigenous Meliaceae, which can be used for comparisons at a continental and global level. Additionally, to identify knowledge gaps in terms of the ethnobotany and chemistry that can be used as a guide for future research work.

## 2. The Ethnobotany and Chemistry of South African Meliaceae

### 2.1. Ethnobotanical Uses

Although there are many uses in woodwork, staining, and construction [36], the traditional and contemporary uses as medicines are also well documented [6,12,13,29,30,37,38,39]. Furthermore, the plants find a place in horticulture for ornamental purposes and shade, as food, as anti-feedants, and for ritual purposes [36]. The most frequently cited medicinal uses of species in Meliaceae are as anthelmintics and antimicrobials. However, they are included in a wide range of applications including as therapeutic interventions in cardiovascular function, and respiratory, urinary, gastrointestinal, dermatological, and oral infections [12,30].

**Figure 1:**
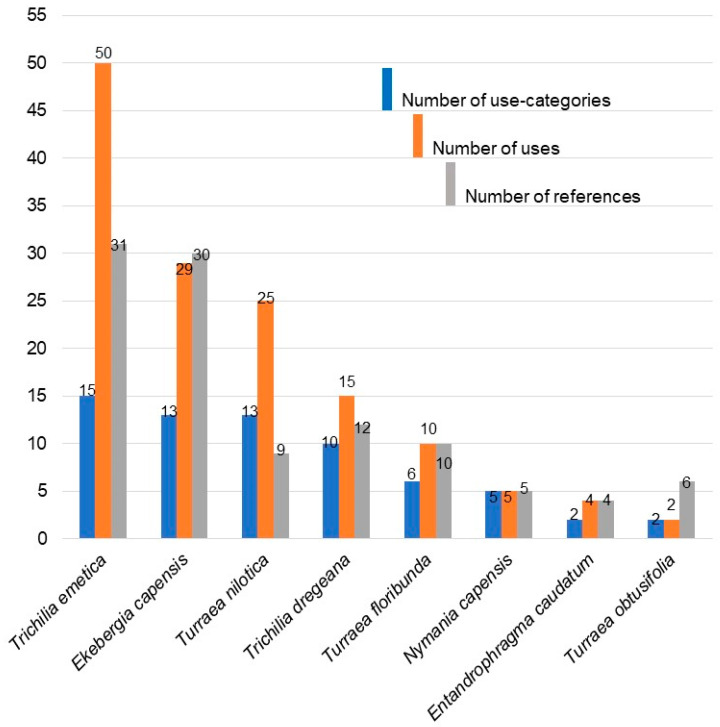
Number of use-categories, uses, and references for each species of South African Meliaceae.

Ethnobotanical uses and prescriptions of South African Meliaceae are reported in Table 1. Although six genera and twelve species are recognised in South Africa, only eight species from five genera have recorded ethnomedicinal uses, giving a total of 85 different ethnomedicinal uses summarised from published records. Out of the functional uses, *Trichilia emetica* Vahl demonstrated the highest utility, followed by *Ekebergia capensis* Sparrm (Figure 1). Both were frequently cited as being used as shade trees and for ornamental purposes, as well as for furniture, timber, and cosmetics (Section 2.1.5).

In the context of strictly therapeutic applications, *E. capensis* represents an important complement to South African *materia medica* [6]. The parts of *E. capensis* used traditionally vary according to end-use, but range from bark, leaf, fruit, root, wood to twig. Such applications include the treatment of epilepsy, malaria, pain, skin ailments, and gastrointestinal, respiratory, cardiovascular, and reproductive problems (Table 1). 

The most commonly used organs are the bark or root bark, administered as a decoction that is boiled in about 2 L of water and taken as an emetic for coughs, heartburn, and respiratory chest complaints [6]. When a poultice is made from the crushed bark, it is combined with flour and water as a caking agent and applied as a skin scrub for use as a topical blood purifying agent for abscesses, boils, and in hot water infusions for pimples [13]. Different parts of the plant could either be used alone or in combination with other species. The bark powder and leaf decoctions are used in the treatment of intestinal worms and epilepsy [13,30,40]. In this regard, approximately 200 mL of the aqueous leaf infusion is drunk as a purgative parasiticide. Furthermore, the bark and root are combined to treat gastritis, dysentery, heartburn, and as an expectorant [6,8,12]. The bark is also used in rituals to guard tribal chiefs against witchcraft and taken orally as a love charm emetic [13].

**Table 1:** The traditional uses of South African Meliaceae. The categories are according to Moffett’s (2010) classification. *NR: Not recorded; A: Afrikaans; E: English; N: Ndebele; NS: Northern Sotho; S: Sotho; Sh: Shona; T: Tsonga; Ts: Tswana; V: Vhavenda; X: Xhoza; Z: Zulu.

Taxa	Local Names	Traditional Use			References
		Medicinal Use	Part Use	Preparation and Administration	
*Ekebergia capensis* Sparrm.	Esseboom, essenhout (A); dogplum, cape ash (E); munyonga, mmidibidi (NS); umNyamatsi (S); mumbafwe (T); nyamaru (Ts); mudouma, muṱobvuma, muzhouzhou (V); umgwenyezinja (X); imanaya, isimanaye, mahlunzidintaba, ronyamati, simanaya, umathunzi wentaba, umathunzini, umgwenyana wezinja, umnyamathi, umthoma, usimanaye, uvungu, (Z)	**Analgesic**			
		Headache	Root	Powdered, charred pulverized roots are sniffed	[12,41,42]
			Leaf	NR	[43]
		Malaria	Root and leaf	Extracts from maceration of crushed roots and leaves are drunk	[44]
			Bark	inner bark is boiled and drunk	[45]
		**Anthelmintic**			
		Worms	Bark and leaf	Bark powder is added to leaf decoction and drunk	[6,46]
		**Antimicrobial**			
		Anthrax	Leaf	Crushed leaf is boiled and drunk	[47,48]
		Venereal diseases	Bark and root	Freshly collected bark and roots are boiled in water and the extract is drunk three times daily	[49]
		**Cardio-vascular**			
		Blood purifier and blood pressure	Leaf and inner bark	Leaf or inner bark is boiled and drunk	[13,45,47]
		Heart ailment	Bark	NR	[13]
		**Cytological**			
		Cancers	Fruits	Fruits are crushed, sieved, and drunk	[50]
		**Dermatological**			
		Abscess, scabies, and acne	Bark	Infusion or maceration of the bark powder is applied	[6,39,51,52]
		Scabies	Root and leaf	NR	[12,41]
		Abscess and boil	Bark	Crushed bark added to flour and water poultices is applied	[13,51]
		Pimples	Bark	Crushed bark in hot water infusion is drunk and used as a wash	[13,51]
		Skin ailments	Leaf	NR	[37]
		**Gastro-Intestinal**			
		Bloody stool	Bark	Bark is macerated with bark of *Diospyros lycioides* Desf. and extract is drunk	[53]
		Emetic and heartburn	Bark and root	Bark or root decoctions are taken as emetics	[6,43,54]
		Gastritis, dysentery, and heartburn	Bark and root	One teaspoon of bark and root powder in a half cup of hot water is taken as tea	[6,12,41,44,55]
		Purgative	Leaf	A cup of leaf infusion is drunk	[13]
		Stomachache	Fruits	Fruits are masticated and swallowed	[50]
		**Gynaecological and Obstetrics; Genital system**			
		Dystocia	Leaf and twig	Leafy twigs mixed with *Indigofera oubanguiensis* Tisser. are taken orally and used as a wash to treat dystocia	[30,56]
		Infertility	Bark	NR	[13]
		**Nervous system**			
		Epilepsy	Bark	Bark infusion is drunk	[57]
		Stress relief	NR	NR	[14]
		**Respiratory system**			
		Chest complaints and coughs	Bark and root	Bark or root decoctions are taken orally	[54]
		Chronic cough	Leaf	NR	[43]
		Cough and respiratory complaints	Bark	Bark decoction with root of *Euclea natalensis* A.DC. is drunk	[54]
		**Trauma**			
		Snakebite	Root and leaf	Extracts from maceration of crushed roots and leaves are drunk	[44]
		**Ethnoveterinary**			
		Tuberculosis	Bark	Crushed bark is boiled and administered orally	[48,58]
		Abortion	Galls	Galls on plant are boiled and administered orally	[59]
		**Magic**			
		Protection	Bark	Bark is used to protect chiefs against witchcraft	[13,60]
		Love	Bark	Bark decoction is drunk as love charm emetics	[13,60]
*Ekebergia pterophylla* (C.DC.) Hofmeyr	Rotsessenhout (A); rock ash (E); maGwedla (S)	No Ethnomedicinal Records			
*Entandrophragma caudatum* (Sprague) Sprague	Bergmahonie (A); mountain mahogany, wooden-banana (E); mophumêna (Ts); munzhounzhou (V)	**Analgesic**			
		Malaria	Bark	NR	[61]
		**Antimicrobial**			
		Gonorrhoea	Root	Root decoction is drunk	[62]
		Genital warts	Fruit	Burnt fruit peels mixed with Vaseline are applied topically	[62]
*Nymania capensis* (Thunb.) Lindb.	Kankerbos, kiepkiepies, klapperbos, klapperbossie, lanternbos, oumeidsbos, oumeidebos, stuipebos, stuipebossie, stinkbossie, ystervarkbos (A); chinese lantern tree, kipkippers, klapper (E)	**Ear, Nose, and Throat**			
		Influenza	Leaf	Leaf infusion is taken	[63]
		**Gastro-Intestinal**			
		Stomach complaints and nausea	Root	Root decoction is taken	[64]
		**Nervous system**			
		Convulsion	Leaf	Leaf decoction	[12,53,63]
		**Trauma**			
		Wound healing	Root	Roasted, pulverized roots are sprinkled on the affected part and can also be mixed with fat into an ointment and applied as a salve	[64]
		**Urinary system**			
		Kidney problem	Root	Root decoction is taken	[64]
					
*Pseudobersama mossam bicensis* (Sim) Verdc.	Valswitessenhout (A); umopho (Z)	No Ethnomedicinal Records			
*Trichilia dregeana* Sond.	Bos Rooi-essenhout, bosrooiessenhout (A); cape mahogany, forest mahogany, forest natal mahogany, white mahogany (E); mutshikili, mutuhu, muuhu (V); umhlakele, umkhuhlu, umkhuhlwa (X), ixolo, umathunzi, umathunzini, umkhuhlu (Z)	**Analgesic**			
		Back pain	Bark	A teaspoon of pulverised bark boiled in a cup of milk is allowed to cool and strained, then half a cup of the extract is taken as an enema in the early morning	[12,13]
		Fever	Root	Root decoction is taken orally	[12,65]
		Toothache	NR	NR	[14]
		**Antimicrobial**			
		Gonorrhoea and Syphilis	Leaf	Handful of leaves is boiled with a handful of *Albizia adianthifolia* leaves in 2 L water and half a cup of the decoction is taken daily	[27,43,53,66]
		Leprosy	NR	NR	[65]
		**Cardio-Vascular**			
		Blood purifier	Bark	Bark is taken as an enema for men	[43]
		**Dermatological**			
		Bruises and eczema	Leaf or fruit	Leaf or fruit poultice is applied topically	[65]
		**Gastro-Intestinal**			
		Bloody diarrhoea	Bark	Bark decoction is taken daily	[53,65]
		Purgative and stomach complaints	Bark or root	Bark infusion is taken as an enema or root decoction is taken orally	[13,65,67]
		**Gynaecological and Obstetrics; Genital system**			
		Abortifacient	Bark	Bark infusion or decoction is taken orally or used as an enema	[65,67]
		**Musculo-Skeletal**			
		Lumbago	NR	NR	[65]
		Rheumatism	Seed	Oil from seed is used for massage	[65]
		**Trauma**			
		Fractures	Seed	Oil from seed is rubbed into scarifications made on fractured limb	[65]
		**Urinary system**			
		Kidney problem	Bark	Bark maceration is taken as an enema	[13,43,53]
		**Ethnoveterinary**			
		Fishing poison	Bark	NR	[8,65,67]
*Trichilia emetica* Vahl	Rooiessenhout (A); natal-mahogany (E); mamba (NS); umkuhlu (Si); ankulu, nkulu (T); mutshikili, mutuhu (V); umkhuhlu (X); umathunzini (Z)	**Analgesic**			
		Back pain	Bark or leaf	Bark or leaf maceration is taken as an enema	[12,13,68]
		Dental care	Twig, trunk, wood, root or flower	Twig, trunk, wood or root is chewed and the crushed flowers is used as a toothpaste	[69]
		Headache	Leaf	Leaf infusion is used to wash the head	[69]
		Malaria	Leaf, bark, and root	Leaf decoction mixed with lemon is drunk or used as a bath for 3–7 days. Bark decoction mixed with honey is also taken orally. Root, stem, and leaf decoction is taken 2–3 times daily for 3 days. Root maceration of *T. emetica*, *Pseudocedrala kotschii* (Schweinf.) Harms and *Nauclea latifolia* Sm. mixed with honey can also be drunk for 10 days	[38]
		**Anthelmintic**			
		Teniasis	Bark	Crushed bark mixed with root of *Securidaca longependonculata* Fresen. is taken orally for 3 days	[38]
		Worm	Bark or root	Bark or root decoction is taken daily for 3 days	[53,70]
		**Antimicrobial**			
		Dysentery	Bark or leaf	Bark or leaf maceration is taken as an enema	[12,13,71]
		Gonorrhoea and syphilis	Bark and leaf	Bark and leaf decoction is drunk	[43,62]
		Leprosy	Root	Root maceration is drunk or used as a bath	[38,42,71]
		**Cardio-vascular**			
		Blood and digestive tract cleanser	Bark	Bark infusion is applied as an enema	[13,43]
		Blood pressure	Leaf	A teaspoon of 1 h decoction of *T. emetica* leaf, *Aloe. marlothii* leaf, and *Hyphaene coriaceae* root is taken orally three times daily	[27]
		**Dermatological**			
		Burns and bruises	Leaf	Hot leaf infusion is applied to the affected part	[69]
		Dermatitis	Leaf and bark	Leaf decoction is used in a steam bath or crushed leaves are applied on the affected part while the powdered bark can also be used for cleansing	[38,72]
		Eczema	Fruit or leaf	Fruit or leaf poultice is applied topically	[12,13,73,74]
		**Ear, Nose, and Throat**			
		Colds and bronchial inflammation	Root	Root decoction is taken orally	[37]
		**Gastro-Intestinal**			
		Abdominal pain	Leaf or root	Crushed leaf or root decoction is used as a bath and taken orally with salt and lemon twice daily	[38]
		Constipation	Bark	50 g of chopped bark is boiled with 50 g of chopped bark of *Spirostachys africana* Sond. in 5 L of water and taken orally	[68]
		Diarrhoea	Bark	Bark infusion is administered anally twice a day	[27]
		Digestive infections	Root	Root decoction mixed with coffee is taken orally for 3 days or decoction mixed with *Cassia sieberiana* DC. root and honey drunk in the morning for 5 days	[38]
		Emetic	Bark or root	Bark maceration or pulverized bark in hot water is taken, root extract can also be taken orally	[12,13,37,46,72,75,76,77]
		Flatulence	Root	Powdered root infusion added to *Acacia nilotica* seed powder is taken	[38]
		Hemorrhoids	Root	Crushed root bark mixed with black pepper and crushed fruit of *Xylopia aethiopica* (Dunal) A. Rich. is taken daily, while the powdered root in salt water is used as an enema	[38]
		Gastric ulcer	Bark	Crushed bark mixed with salt and ginger is added to porridge and taken twice daily	[38]
		Hernia	Root	Crushed root is added to porridge and taken orally	[38]
		Inflamed anus	Bark	Pulverized bark puffed into the anus	[30]
		Jaundice	Root	Root decoction is taken daily for 3 days, chopped roots are also mixed with honey and used as a bath	[38,70]
		Laxative	Bark	Bark is mixed with eggs and taken orally to clean the stomach	[78]
		Purgative	Bark	Bark infusion or decoction is used as an enema or taken orally	[53,67,76]
		Stomach complaints	Bark	Bark infusion or decoction is taken orally or administered as an enema	[43,53,54,67]
		**Gynaecological and Obstetrics; Genital system**			
		Abortifacient	Bark	Bark infusion is taken	[67,71]
			Bark and root	One handful of crushed bark and root in a litre of milk and Coca Cola mixture is boiled for 15 min and taken orally	[27]
		Breast pain	Leaf	Leaf decoction is used to bath	[38]
		Dysmenorrhoea	Leaf	Leaf decoction added to *Tamarindus indica* L. or lemon is taken orally for 7 days	[38,79]
		Fertility	Leaf	Leaf decoction with *Combretum molle* R. Br. ex G. Don is taken orally	[38]
			Bark	Crushed bark mixed with the same amount of Gymnosporia *senegalensisa* root is boiled and decoction is used as an enema	[27]
		Labour pain	Leaf	Hot leaf decoction is used to massage the belly of a woman during labour to ease pain until she delivers	[27]
		Sterility	Root	Powdered root with ginger and salt is taken as a porridge	[38]
		**Musculo-Skeletal**			
		Lumbago	Bark or leaf	Bark or leaf maceration is taken as an enema	[12,30]
		Paralyses	Root	Crushed root is mixed with porridge and taken orally	[38]
		Rheumatism	Seed	Oil from boiled seeds is taken orally and rubbed topically	[8,12,13,46,75]
		**Opthalmic**			
		Eye infection	Leaf and bark	Leaf and bark decoction is used for eye cleansing for 2 days	[38]
		**Respiratory system**			
		Cardiac problems	Leaf	Leaf decoction is taken orally	[80]
		Chest pain	Leaf	Leaf decoction is used in a steam bath or rubbed on the chest	[38]
		Cough	Bark and root	Bark and root decoction is drunk	[81]
		Pneumonia	Root or leaf	Root or leaf decoction is taken orally or used as a bath for 12 days	[37,38]
		**Trauma**			
		Fracture	Seed	Oil from the seed is rubbed into incisions on broken limbs and baked pulverized root of *Sideroxylon inerme* L. is applied	[8,12,13,82]
		Stiffness or sprains	Bark	Bark extract is applied topically	[69]
		Wound	Seed	Oil from the seed is applied to prevent infection from maggots	[37]
		**Urinary system**			
		Kidney problem	Bark	Bark decoction is administered as an enema	[43]
		Renal ailments	Bark	Bark decoction is taken	[13]
		**Ethnoveterinary**			
		Fishing poison	Bark	NR	[67]
		**Magic**			
		Burial rituals	Leaf	Leaves are worn during burial rituals	[54]
*Turraea floribunda* Hochst.	Kanferfoelieboom (A); honeysuckle-tree, wild honeysuckle-tree (E); umdlozana, inkunzane (Si); umhlatholana, umlahlana (X); umadlozane, umlulama, ubhukulo (Z)	**Dermatological**			
		Abscesses	Root	Root decoction is taken orally	[30]
		**Gastro-Intestinal**			
		Ascites	Root	Root maceration is taken orally	[12,13]
		Emetic	Bark	Bark extract is taken orally	[8,37]
		Purgative	Bark and root	Bark and root decoction are taken orally	[37]
		**Musculo-Skeletal**			
		Rheumatism	Root	Root maceration is taken orally	[8,12,13,75,76]
		**Respiratory system**			
		Cardiac problems	Root	Root maceration is taken orally	[12,13,47,75,76]
		Cough	Root	Root decoction is taken orally	[37]
		**Urinary system**			
		Urethral infection	Bark	Bark decoction is taken orally 1-3 times daily	[83]
		**Magic**			
		To induce a state of trance	Bark	Bark infusion is taken orally	[30,75,76]
		Protection from bad dreams	Bark	NR	[76]
*Turraea nilotica* Kotschy and Peyr.	Bushveld honeysuckle-tree, lowveld honeysuckle-tree, miombo honeysuckle-tree, small mahogany (E) isidlamvundala (N); chipindura, chirambagavakava, chitsvimbovarisa, chitunguru, mudyakuwe, mukondanyoka, muzaramhanga (Sh)	**Analgesic**			
		Headache	Root or leaf	Root decoction or leaf infusion is taken orally	[46,67]
		Toothache	Root	Root decoction is used as a mouthwash	[76,84]
		**Anthelmintic**			
		Ascariasis	Root	Root decoction is taken orally	[30]
		**Antimicrobial**			
		Gonorrhoea	Root	Root decoction is taken orally	[81]
		Venereal diseases	Root	Root infusion is taken orally	[8,67]
		**Dermatological**			
		Abscesses	Root	Root decoction is taken orally and applied as a compress	[30]
		**Gastro-Intestinal**			
		Abdominal pain	Leaf or root	Leaf decoction or root infusion is taken orally	[8,67,85]
		Constipation	Root	Root bark decoction is taken orally	[8,81]
		Diarrhoea	Leaf or root	Leaf or root decoction is taken orally or pulverized root is added to porridge	[8,67,85]
		Indigestion	Root	Root decoction is taken orally	[37]
		Schistosomiasis or hernia	Root	Root infusion mixed with honey is taken orally	[81]
		**Gynaecological and Obstetrics; Genital system**			
		Aphrodisiac	Root	Powdered root mixed with beer or porridge is taken	[67]
		Dysmenorrhoea	Root	Powdered root mixed with porridge is taken	[8,67]
		Prevent abortion	Root	Root infusion is taken orally	[67]
		Sterility	Root	Root decoction is taken orally	[81]
		Nervous system			
		Dizziness	Leaf	Leaf infusion is taken orally	[67]
		Epilepsy	Root	Powdered root mixed with porridge is taken	[8,67]
		**Opthalmic**			
		Eye problems	Leaf	Leaf paste is applied to the eyelids	[67]
		**Respiratory system**			
		Dyspnea	Root	Powdered root mixed with porridge is taken orally	[67]
		Pneumonia	Root	Powdered root is rubbed into scarification on the painful area, root infusion is taken orally, and smoke from the burnt roots is inhaled	[8,67]
		**Trauma**			
		Snakebite antidote	Root	Burnt root ashes are applied to the bite	[67]
		Wound	Root	Root scrapings are applied topically	[30]
		**Urinary system**			
		Dysuria or rectal prolapse	Root	Root bark decoction is taken orally	[67]
		**Ethnoveterinary**			
		Anthelminthic for dogs	Root	Root infusion is administered orally	[67]
		**Magic**			
		To calm the insane	Leaf	Leaf infusion is administered orally and smoke from burnt leaves is inhaled	[67]
*Turraea obtusifolia* Hochst.	Kleinkanferfoelieboom (A); small honeysuckle tree, lesser honeysuckle tree, (E); amzulu, ikhambi-lomsinga, ikunzi, inkunzi, inkunzi-embomvana, umhlatholana, uswazi (Z)	**Gastro-Intestinal**			
		Stomach and intestinal complaints	Leaf, bark and root	Hot water maceration of leaf, bark or root is given as an enema and also mixed with porridge to be taken orally	[13,54,75,76,86]
		**Ethnoveterinary**			
		Wounds in livestock	Leaf	Crushed leaves are applied topically on the affected part	[87]
	
*Turraea pulchella* (Harms) T.D.Penn.	No Ethnobotanical Record				
*Turraea streyi* F. White and Styles	No Ethnobotanical Record				

#### 2.1.1. Categories of Medicinal Uses

**Figure 2:**
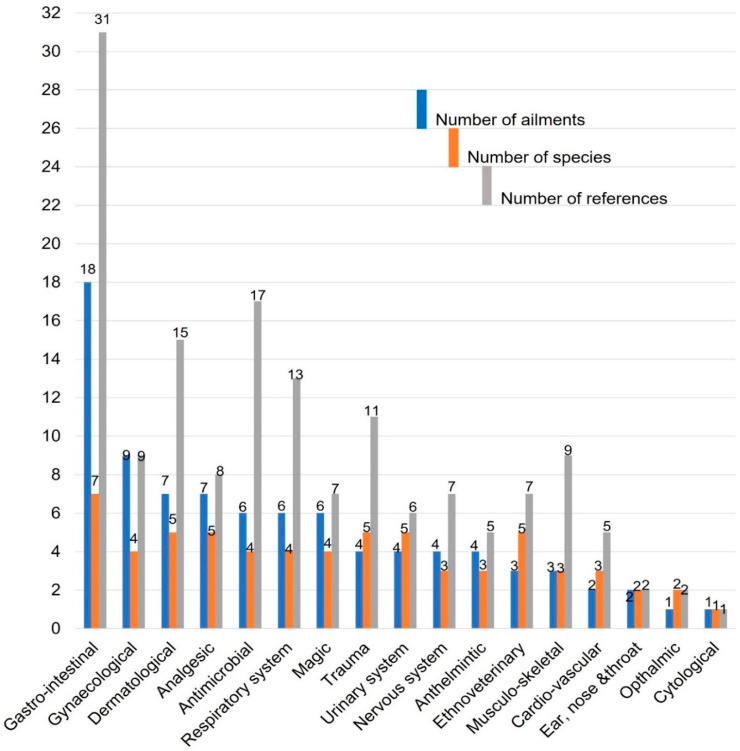
Number of ailments in each category, species used in the treatment of each category of ailment, and references for South African Meliaceae.

Remedies made from the South African Meliaceae are used to treat a wide variety of medical conditions in humans, as well as for ritual purposes. They are also used in ethnoveterinary treatments. Most of the species had more than one therapeutic use, with *T. emetica* having the highest number of uses and categories (50 and 15, respectively), followed by *E. capensis* (29 and 13, respectively). The lowest number of uses and categories was recorded against *T. obtusifolia* (Figure 1). The highest number of citations for ethnomedicinal uses was recorded against *T. emetica* (31) followed by *E. capensis* (30), *T. dregeana* (12), *T. nilotica* (9), *T. obtusifolia* (6), *N. capensis* (5), and *E. caudatum* (2) (Figure 1). Eighty-seven different ailments grouped into 17 major categories which are gastro-intestinal; gynaecological and obstetrics; dermatological; analgesic; antimicrobial; respiratory system; magic; trauma; urinary system; nervous system; anthelmintic; ethnoveterinary; cardio-vascular; ear, nose and throat; ophthalmic; and cytological were recorded in this study (Figure 2). Most of the species of South African Meliaceae are mostly used in the treatment of gastro-intestinal ailments followed by gynaecological and obstetrics related ailments (Figure 2). However, there was no ethnomedicinal record found for four of the species (*E. pterophylla*, *P. mossambicensis*, *T. pulchella,* and *T. streyi*).

An example of the use of *E. capensis* in multi-therapeutic combinations with other species is the decoction that is made from a combination of *E. capensis* leafy twigs and *I. oubanguiensis*, which is taken orally and used as a wash to treat dystocia [30]. Bryant [54], also described how the bark decoction of *E. capensis* is mixed with roots of *E. natalensis*, to be taken orally to treat respiratory problems. More examples of species combinations in multi-therapeutics with *E. capensis* are provided in Table 1. Preparation of herbal remedies using more than one plant species can be attributed to the synergistic or additive effects that could occur during the treatment [88].

In contrast to *E. capensis*, *E. caudatum* has minimal presence in South African *materia medica*. The root decoction is being used as a remedy for gonorrhoea, while the burnt fruit is mixed with Vaseline and applied topically to treat genital warts [62]. This is not the only record of dry heating/burning as a *materia medica* modality. 

In some applications, *N. capensis* processing involves a roasting step, i.e., according to Von Koenen [64] the root is roasted, pulverized, and applied topically to treat wounds and relieve knee pain. However, medical uses of *N. capensis* broaden to include a root decoction taken orally to treat kidney and stomach complaints, as well as nausea. The leaf decoction is also taken orally as a herbal remedy for convulsion [63,89].

*Trichilia dregeana* is an important medicinal plant with all of the parts used traditionally [55]. It is used as a herbal remedy for the treatment of syphilis, bloody diarrhoea, skin diseases, rheumatism, as an abortifacient, blood cleanser, and as fish poison (Table 1). The bark infusion or maceration is used as an enema for the treatment of kidney problems, bronchial inflammation, skin diseases, as well as general cleaning [13,43,52]. The bark is eaten as a purgative or for procuring abortion and also as a fish poison [67]. The leaf decoction is taken orally as a herbal remedy for syphilis [13,66]. 

Similar to *E. capensis*, species in *Trichilia* are also strongly represented in the ethnobotanical tradition of South African people. *Trichilia emetica* is a multipurpose tree that is widely distributed throughout Africa, meaning it is not exclusively a South African medicine [38]. All plant parts, the leaf, twig, bark, flower, wood, root, and fruit of *T. emetica* are used (Table 1). It is used as a purgative, an antipyretic, antiepileptic, and antimalarial agent [72]. The twig, trunk, wood, and root are chewed as herbal remedy for dental care [30]. The powdered bark is taken orally as a remedy for infertility and also to ease labour pain [27,38]. The bark decoction or infusion is also used to treat various ailments including dysentery, gastrointestinal problems, breast pains, back pains fever, malaria, and as a purgative (Table 1). The bark decoction is used by the Xhosa tribe as an enema to treat kidney problems [12]. 

In other applications, a hot leaf infusion of *T. emetica* is rubbed on the affected part to treat burns while the leaf decoction is taken orally as a remedy for dysmenorrhoea and syphilis [13,38]. The leaves are also used as a poultice for wound healing, skin problems, and contusions [29,52]. The leaves can also induce drowsiness or sleep at night when placed in the bed [38]. The root decoction is used as a herbal remedy for colds and bronchial inflammation, chest pain, fever, pneumonia, jaundice, gastrointestinal infections, and sexually transmitted diseases (Table 1). The fruit is used as a herbal remedy for eczema [13,82]. Pulverized seeds of *T. emetica* are boiled and the oil is rubbed on the affected part to treat rheumatism and leprosy [46]. However, the combination of two or more organs of *T. emetica* can also be used as a herbal remedy (Table 1) that is allegedly more potent than the individual parts. The decoction of stem, root, and bark is taken orally as a herbal remedy for whooping cough and ulcers [38,81]. The leaf and bark decoction of *T. emetica* is rubbed on the eye to treat an eye infection [38]. 

Similar to other taxa in Meliaceae, the bark and root of *T. floribunda* is used as a remedy for a broad range of ailments (Table 1). It is boiled in water and taken orally as an emetic and herbal remedy for urethral infection [37,54,83]. A bark infusion of *T. floribunda* is also taken orally to induce a state of trance prior to rituals [75]. The root decoction is taken orally as a remedy for cough and hardened abscess [37], while the root maceration is taken orally to treat rheumatism, cardiac problems, ascites, and dropsy [8,13]. The root and bark decoction are taken orally as a purgative [12,37]. 

The root decoction of *T. nilotica* is taken orally as a remedy for headaches, hardened abscess, indigestion, gonorrhoea, sterility, dysuria, jaundice, as a poison antidote, and against intestinal worms [30,67,70,81]. The root decoction is used as a mouth wash for toothache and the ash of the burnt root is applied topically as a snakebite antidote [30,84]. The root infusion is taken orally to treat venereal diseases, abdominal pain, constipation, inflammation of navel cord, and to prevent abortion [8,30,67]. The root infusion is taken orally with honey to treat schistosomiasis, hernia, and bilharziasis, while in ethnoveterinary medicine, the root infusion is used as an anthelmintic for dogs [8,81]. The root powder is taken orally in beer or porridge as an aphrodisiac, it could also be used as a remedy for dysmenorrhea, epilepsy, and dyspnoea [67]. The leaf decoction is taken orally as a remedy for abdominal pain and diarrhoea, while the leaf infusion is taken orally as a remedy for dizziness [30,37,85]. The leaf infusion of *T. nilotica* is taken orally and the smoke from burnt leaves is inhaled to calm an ‘insane person’, while the leaf paste is applied to the eyelid to treat eye problems [67].

The leaves, bark, and root bark of *T. obtusifolia* are macerated together in hot water and taken with porridge as a remedy for stomach and intestinal complaints [86]. Due to the diverse use of *E. capensis*, *T. dregeana*, *T. emetica*, *T. floribunda*, and *T. obtusifolia* as herbal remedies, the bark is sold in informal herbal medicine markets as traditional medicines in Gauteng and KwaZulu-Natal provinces in South Africa [90].

#### 2.1.2. Categories of Uses

Remedies made from South African Meliaceae are used to treat a wide variety of medical conditions in humans as well as for ritual purposes. They are also used in ethnoveterinary treatments. All of the species had more than one therapeutic use, with *T. emetica* having the highest number of uses and categories (50 and 15, respectively), followed by *E. capensis* (29 and 13, respectively) and the lowest number of uses and categories was recorded against *T. obtusifolia* (Figure 1). The highest number of citations for ethnomedicinal uses was recorded against *T. emetica* (31) followed by *E. capensis* (30), *T. dregeana* (12), *T. nilotica* (9), *T. obtusifolia* (6), *N. capensis* (5), and *E. caudatum* (2) (Figure 1). Eighty-seven different ailments were grouped into 17 major categories including gastro-intestinal; gynaecological and obstetrics; dermatological; analgesic; antimicrobial; respiratory system; magic; trauma; urinary system; nervous system; anthelmintic; ethnoveterinary; cardio-vascular; ear, nose, and throat; opthalmic; and cytological were recorded in this study (Figure 2). South African Meliaceae are mostly used in the treatment of gastro-intestinal ailments followed by gynaecological and obstetrics related ailments (Figure 2). However, there was no ethnomedicinal record found for four of the species (*E. pterophylla*, *P. mossambicensis*, *T. pulchella*, and *T. streyi*). 

#### 2.1.3. Plant Parts Used

**Figure 3:**
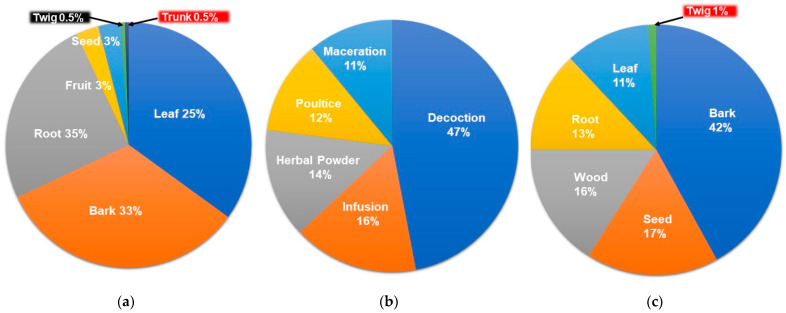
(**a**) Percentage of different plant parts of South African Meliaceae reported to be used in ethnomedicine; (**b**) different dosage forms of herbal remedies of South African Meliaceae reported in the literature; (**c**) plant parts of South African Meliaceae from which compounds were reported to be extracted.

The plant parts of South African Meliaceae used in making herbal remedies were the root, bark, leaf, fruit, seed, twig, and trunk. Roots (35%), followed by barks (33%) and leaves (25%), were the most frequently used plant parts in preparation of the recorded herbal remedies (Figure 3a). Several studies reported roots to be more effective than other herbal plant parts. Hence, these were most frequently sourced [5,91,92,93]. This practice can also be linked to the scientific reasoning that roots and other underground parts contain high concentrations of bioactive compounds [94]. However, harvesting of roots for medicinal purposes is not sustainable as it threatens the existence of many medicinal plants which could lead to depletion of the plants. It is well documented by conservationists that medicinal plants mostly sourced for their root parts and bark are likely to be the most threatened by over-exploitation [95].

#### 2.1.4. Mode of Herbal Preparation

Decoction (47%) was the most common mode of preparation recorded followed by infusion (16%), direct use as herbal powder (14%), poultice (12%), and maceration (11%) (Figure 3b). Decoction (boiling of the plant material) has been reported to be the most commonly used method of preparation in herbal medicine as it is believed that boiling extracts all of the potential bioactive compounds from the plant [96,97,98,99,100]. Moreover, decoction was reported to be the most common method of herbal preparations in South Africa [6]. Most of the remedies were administered orally followed by topically in the case of wound and skin infections, while some are sniffed into the nose (Table 1).

#### 2.1.5. Other Uses

South African Meliaceae have also been reported to be useful for other purposes apart from medicinal uses. *Ekebergia capensis, E. pterophylla, Entandrophragma caudatum, T. dregeana, T. emetica, T. floribunda,* and *T. obtusifolia* are used as ornamental plants in gardens and on roadsides to create shade, wind breaks, and soil conservation (Table 2). The timber of *E. capensis, E. caudatum, P. mossambicensis,* and *T. dregeana* are mostly sought after by the furniture industry since they are soft, easy to work with, and durable (Table 2).

The wood of *E. capensis*, *P. mossambicensis, and T. nilotica* are used for charcoal and firewood for cooking (Table 2). Birds feed on the fleshy part of the fruit of *E. capensis* and the leaves are used as a fodder for domestic stock and game [12,40]. Edible caterpillars gathered from *E. capensis* are eaten as food by the Vhavenda [43,101]. *Nymania capensis* is used as a garden plant and also as a source of forage for goats [89]. The seeds of *T. dregeana* and *T. emetica* are known for their high fat content, hence the fat is used in soap making, as a body ointment, as polish, hair oil, and in cooking [43,75,103]. The seed arils of *T. dregeana* and *T. emetica* are cooked as vegetables or crushed for the milky juice which is taken with side dishes or as a drink [8,101]. In northern KwaZulu-Natal, the wood of *T. dregeana* is used to carve birds and animals which are sold along roadsides [8].

The wood of *T. emetica* is used to carve meat dishes, bowls, spoons, head rests, and animal carvings in Maputaland [4]. The leaves of *T. emetica* are eaten by wild animals and also worn in burial rituals by the Zulu [13,82]. The VaVhenda people in South Africa use the wood to construct the frame of an African traditional musical instrument (‘mbila’), while oil from *T. emetica* is applied on the instrument to soften the animal skin used for the instrument [82]. Saka and Msonthi [106] reported the juice from *T. emetica* seeds mixed with other edible plants to be used as multivitamins in cases of malnutrition.

The wood of *T. floribunda* is used for making traps, while stems of *T. nilotica* are used for handcrafts [107]. *Turraea obtusifolia* can also be used as a container plant in landscape design [76].

### 2.2. Reported Active Compounds

Several limonoids and other secondary metabolites of appreciable biological activities have been reported in South African species of the Meliaceae. The extracted compounds and parts extracted are represented in Table 3. Chemical studies for 10 out of the 12 South African Meliaceae were found. The two species that had no records are *T. streyi* and *T. pulchella*. 

The compounds are classified according to nine categories of chemical class which are: (1) Limonoids, (2) triterpene, (3) coumarin, (4) glycoflavonoid (glycoside), (5) phenolic aglycone, (6) sterol (phytosterol), (7) pregnane, (8) protolimonoid, and (9) sesquiterpene. The highest reported chemical classes were limonoids followed by triterpenes and sterols. However, sterols are common in higher plants. On the other hand, a significant number of triterpenes, coumarins, and limonoids are described in South African species for the first time and their naming is etymologically related to the genus or species. Unsurprisingly, the richest diversity of new metabolites include a heterocyclic moiety in the form of a lactone, coumarin or furan.

Novel types of limonoid of the ekebergolactone class were first described from a species of *Ekebergia* and received the name capensolactones, which were isolated among others (Table 3) from the leaves, seeds, and stem bark of *E. capensis* [31,33,108,109]. From *E. pterophylla,* more new limonoids were described and given initials taken from the genus and species name (EP1-EP6), which are closely related to methyl angolensate and similar to those of other *Ekebergia* species [110,111]. Furthermore, complex limonoids referred to as *Nymania* 1-4 were isolated from the bark of *N. capensis* [112], reiterating the etymological genesis.

All of the species in the current study, except one, were rich sources of new limonoids, often described nowhere else in the world. While no limonoids were reported from *P. mossambicensis,* the nine other species investigated so far constitute valuable sources of these compounds [113,114,115]. The limonoids of *E. caudatum* were named phragmalins after the genus [116]. From seeds of *T. dregeana*, the limonoids include *dregeana* 1–4 [117]. Several limonoids from other South African Meliaceae were assigned in the stem bark of *T. emetica,* and previously undescribed compounds including trichirokin and trichilins A-G [24,118]. Then, in the genus *Turraea* several limonoids were also reported, such as turraflorins A–I and the floribundins A–F (names assigned in the current review, Table 3) from the root of *T. floribunda*, nilotin from the root and stem bark of *T. nilotica* [119,120] or prieurianins from seeds of *T. obtusifolia* [121,122,123].

The limonoid class, per se, was first isolated from citrus, typically from leaves, fruit, and peel of lemons, limes, oranges, pomelos, grapefruits, bergamots, and mandarins (Manners 2007; Hamdan 2011; Wang 2016). Citrus limonoids represent the traditional limonoid that is known as limonin or its derivatives, which occur in aglycone and glycoside forms [124]. They are distinctly different from the ekebergolactone class of limonoid (that is a pentaneotriterpenoid) represented strongly in South African Meliaceae. These include *Nymania* limonoids and capensolactones. Therefore, it is expected that a functional overlap is minimal in the context of biology. 

A third category of limonoids, ekebergins 1–10 from *E. capensis* are triterpenes and have demonstrated anti-plasmodial activity in an in vivo mouse model, giving moderate parasitemia suppression [33]. However, the ekebergins have a structural feature that classifies them as nortriterpenes, but conveys a structure that is between the traditional limonoid and a triterpene. In this regard, some researchers classify them as limonoids, whilst others call them nortriterpenes [125].

The protolimonoids are also of triterpenoid origin but are classed as steroids. Protolimonoids were isolated and assigned as new compounds from the root and stem bark of *T. nilotica* and named according to the species as niloticin and dihydroniloticin [119,120]. Melianone type protolimonoids were isolated and identified from *E. caudatum* [113,114,115] and *T. obtusifolia,* which also had turraeanthin [121,122,123]. 

The species that expressed the highest steroid composition was *T. emetica*, which produced an extract comprising C21 steroids known as 17β-ethylandrostane derivatives or simply pregnanes. Nine pregnanes are known thus far from *T. emetica* [126] and one from *E. capensis* [33]. The pregnanes are agonists of the nuclear pregnane x receptor, which controls the elimination of toxins from the body by xenobiotic monooxygenase metabolism (cytochrome P450 3A) [127]. Activation can cause a variety of effects that include promotion of elimination of toxins that have similar structures. However, drug-drug interactions can also result, preventing co-administered therapies from being metabolised. The net outcome is an increase in drug half-life for some types of xenobiotics, which can be positive in terms of prolonging efficacy effects, but negative by augmenting the risk of toxicity [128].

South African Meliaceae also express several common triterpenes of the oleanane type and common sterols such as β-sitosterol. For example, the type, as well as common triterpenes β-amyrin, β-amyrone, oleanonic acid, lupeol, and other common sterols were reported from the wood and bark of *E. pterophylla* [108]. Ergosterols were isolated from twigs and leaves of *P. mossambicensis* [129], cycloarten-diol triterpenes from the leaves of *T. dregeana* [130], and sterols from the stem bark of *T. emetica* [24]. The common triterpene oleanolic acid and its derivatives from *E. capensis* demonstrated cytotoxicity against cancer cell lines and moderate antiplasmodial activity that may be related to the cytotoxic activity [31]. However, novel acyclic triterpene derivatives of cosatetraene or squalene are also reported in *E. capensis.* These hydroxylated structures are atypical in that they are tail-to-tail sesquiterpenes that demonstrate noteworthy antiplasmodial activity comparable to the ekebergin limonoid mentioned previously [33]. Some sesquiterpenes have also demonstrated these biological effects. Kurubasch aldehyde is another antiplasmodial terpene isolated from *T. emetica*, which is a hydroxylated humulene that is a potent inhibitor (IC_50_ 7.4 μM) of the S180 cancer cell line and demonstrates a modest anti-protozoal effect [131].

South African Meliaceae are also a reservoir of rare coumarins. The pterophyllin 1-5 series was isolated from the wood and bark of *E. pterophylla* [132]. These chemical species belong to the group of furocoumarins. The pterophyllins are moderate antifungal compounds which are active against fruit pathogens [133]. Coumarins were also reported in *E. capensis*, one of which is ekersenin. This compound was first described in *E. senegalensis* but is now known to be widespread in African Meliaceae. Several derivatives of ekersenin were isolated from the stem bark, wood, and root of *E. capensis* [31,32,33,34,35]. While the cytotoxic effects against cancer cell lines have not yet been tested, it will be a worthy undertaking since a related limonoid demonstrated potent inhibition (IC_50_ 6.8 μM) against the A2780 cell line [134], although the authors did not specify the mechanism nor did they screen against healthy cells, hence toxicity was not determined.

**Table 3:** Isolated compounds extracted from various parts of South African Meliaceae.

Plant.	Compound	Part Extracted	References
*Ekebergia capensis*	**Limonoids**		
	Capensolactones 1-3	Seed	[108]
	Methyl 3α-hydroxy-3-deoxy angolensate	Seed	[108]
	Ekebergin	Seed	[109]
	Ekebergins C1-C3	Bark	[33]
	7-Deacetoxy-7-oxogedunin	Bark	[33]
	Methylangolensate	Bark	[33]
	Mexicanolide	Bark	[33]
	Proceranolide	Leaf and bark	[31,33]
	Swietenolide	Bark	[33]
	**Triterpenes**		
	3,11-Dioxoolean-12-en-28-oic acid	Bark	[33]
	Ekebergin A	Bark and root	[31,33]
	Ekebergins D1-D5	Bark	[33]
	Melliferone	Bark	[33]
	7-Acetylneotrichilenone	Bark	[33]
	Lupeol	Bark	[32]
	2-hydroxymethyl-2,3,22,23-tetrahydroxy-6,10,15,19,23-penta methyl-6,10,14,18-tetra cosatetraene	Bark	[31,33,34]
	2,3,22,23-tetrahydroxy- 2,6,10,15,19,23-hexamethyl-6,10,14,18-tetracosatetraene	Bark and wood	[31,33,34,35]
	3-Epi-oleanolic acid	Bark, root, and wood	[31,32,33,34,35]
	3-Oxo-12β-hydroxy-oleanan-28,13β-olide	Bark and root	[33]
	Oleanolic acid	Bark, root, and wood	[31,32,33,34,35]
	**Coumarins**		
	Ekersenin	Bark	[33,135]
	4,6-Dimethoxy-5-methylcoumarin	Bark	[33]
	7-Hydroxy-6-methoxycoumarin	Wood	[35]
	**Glycoflavonoids**		
	kaempferol-3-O-β-D-glucopyranoside; quercetin-3-O-β-D-glucopyranoside	Leaf	[31]
	**Phenolics**		
	Atraric acid	Bark	[32]
	**Sterols**		
	β-sitosterol	Bark and wood	[32,35]
	β-sitosterol oleate; β-sitosterol palmate	Bark	[32]
	**Protolimonoid**		
	Ekebergin B	Bark	[33]
	**Pregnane**		
	(Z)-volkendousin	Bark	[33]
*Ekebergia pterophylla*	**Limonoids**		
	Ekebergin	Seed	[110]
	Ekebergolactones and prieurianin	Seed	[110]
	EP1-EP6	Seed	[111]
	**Coumarins**		
	Pterophyllins 1 and 2	Bark	[108]
	Pterophyllins 3-5	Wood	[108]
	**Triterpenes**		
	Lupeol	Leaf	[108]
	Oleanonic acid; β-amyrin; and β-amyrone	Bark	[108]
	**Sterols**		
	β-sitosterol	Bark	[108]
	β-sitosteryl acetate	Bark	[108]
	**Phenolics**		
	Atraric acid	Bark	[108]
*Entandrophragma caudatum*	**Limonoids**		
	Phragmalin; phragmalin 3,30-diisobutyrate; phragmalin 3-isobutyrate-30-propionate; Entandrophragmin B (12α-acetoxyphragmalin 3-nicotinate-30-isobutyrate)	Seed	[116]
	Bussein A and B; entandrophragmin	Wood	[113]
	**Protolimonoids**		
	3α–turreanthin; melianone	Wood	[114]
*Nymania capensis*	**Limonoids**		
	*Nymania* 1-4; Prieurianin	Bark and Wood	[112]
*Pseudobersama mossambicensis*	**Sterols**		
	Ergosta-5,24(28)-diene-3β, 7α-diol; 24,28-epoxyergost-5-ene-3β, 7α-diol; and ergost-5-ene-3β,7 α,24,28-tetraol	Twig and leaf	[129]
*Trichilia dregeana*	**Limonoids**		
	*Dregeana*-5; dregeanin; and 12-(2′-deacetyl)-dregeanin	Stem	[136]
	*Dregeana* 1-4; hispidin C	Seed	[117]
	**Sterol**		
	Cycloart-23-ene-3β,25-diol	Leaf	[130]
*Trichilia emetica*	**Limonoids**		
	Trichirokin	Stem	[24]
	Rohituka	Stem	[24,118]
	Trichilin A; trichilin B; and 7-acetyltrichilin A	Stem	[137]
	Trichilin C; trichilin D; and trichilin G	Stem	[138]
	Trichilin F and trichilin G	Stem	[139]
	1-acetyltrichilin; Tr-A; Tr-B; Tr-C	Stem	[140]
	*Dregeana*-4; rohituca-3; rohituca-5; rohituca-7; and *Nymania*-1	Stem	[118]
	**Sesquiterpenes**		
	Kurubasch aldehyde	Leaf	[131]
	**Triterpenes**		
	Methyl-1(*S*),23(*R*)-diacetoxy-7(*R*),24,25-trihydroxy-20(*S*)-21,24-epoxy-3,4-*seco*-apotirucall-4(28), 14(15)-dien-3-oate	Stem	[118]
	**Pregnane**		
	1-methoxy-pregnan-17(*R*)-1,4-dien-3,16-dione; 1-methoxy-pregnan-17(*S*)-1,4-dien-3,16-dione; 2,3-seco-pregnan-17(*S*)-2,3-dioic acid-16-oxo-dimethyl ester; 2,3,16-trihydroxy-5-pregnan-17(*R*)-20-yl acetate; 1-methoxy-androstan-1,4-dien-3,16-dione; 2,3-seco-androstan-2,3-dioic acid-16-oxo-dimethyl ester; 3-methoxycarbonyl-2,3-seco-androstan-3-oic acid-16-oxo-2,19-lactone; 2,3,16,20-tetrahydroxy-5-pregnane; 2,3-dihydroxypregnan-16-one	Root	[126]
	**Phenolics**		
	Benzoic acid; protocatechuic acid	Stem	[24]
	**Coumarin**		
	Scopoletin	Stem	[24]
	**Sterols**		
	Ergosta-5,24(28)-diene-3*S*,16*S*,20*S*-triol; *β*-sitosterol; stigmasterol; and *β*-sitosterol-3-*O*-*β*-D-glucopyranoside	Stem	[24]
*Turraea floribunda*	**Limonoids**		
	Floribundin A (11β-acetoxy-3,7-diacetyl-4α-carbomethoxy- 12α-isobutyryloxy-28-nor-1-tigloyl-havanensin);	Root	[141]
	Floribundin B (28-nor-4α-carbomethoxy-11β-acetoxy-12α-(2-methylbutanoyloxy)-14,15-deoxyhavanensin-1,7-diacetate); Floribundin C (28-nor-4α-carbomethoxy-11β-hydroxy-12α-(2-methylbutanoyloxy)-14,15-deoxyhavanensin-1,7-diacetate); Floribundin D (2218-nor-4α-carbomethoxy-11β-acetoxy-12α-(2-methylbutanoyloxy)-14,15-deoxyhavanensin-1-acetate); Floribundin E (28-nor-4α-carbomethoxy-7-deoxy-7-oxo-11β-acetoxy-12α-(2-methylbutanoyloxy)-14,15-deoxyhavanensin-1-acetate)	Root	[142]
	Havanensinoids 2-4	Root	[143]
	Floribundin F (1α,7α-12α -triacetoxy-4α -carbomethoxy-11β-(2-methylpropanoyloxy)-14β,15β -epoxyhavanensin)	Bark	[121]
	Turraflorins A, B, and C	Seed	[144]
	Turraflorins A and B; turraflorins D-I	Seed	[112]
	14,15-deoxytoonacilin	Seed	[145]
	Toonafolin A and B	Seed	[146]
	**Sterols**		
	Stigmasterol and sitosterol	Wood	[146]
*Turraea nilotica*	**Limonoids**		
	Nilotin	Root	[119]
	Mzikonone; azadirone; 12α-acetoxy-7-deacetylazadirone; 1α,3α-diacety-7α-tigloyvilasinin	Root	[147]
	**Protolimonoids**		
	Niloticin; hispidol B; piscidinol A; toonapubesin F	Bark	[147]
	Niloticin; dihydroniloticin; and piscidinol	Bark	[120]
	**Sterols**		
	Sitosterol-3-O-β-D-glucopyranoside acetate; stigmasterol-3-O-βD-glucopyranoside acetate; and sitosterol-3-O-β-D-glucopyranoside	Leaf	[147]
*Turraea obtusifolia*	**Limonoids**		
	*Nymania*-1	Seed	[122,148]
	Prieurianin and rohitukin	Seed	[123]
	Prieurianin	Whole plant	[121]
	**Protolimonoids**		
	7-deacetylglabretal-3-acetate	Wood	[149]
	Melianone; *Turraea*nthin	Seed	[148]
	Melianodiol; melianotriol; and 7,8-dihydro*Turraea*nthin 3-acetate	Wood	[146]
	Melianone; sepalin-F	Leaf	[146]

#### 2.2.1. Class of Isolated Compounds and Parts Extracted

**Figure 4:**
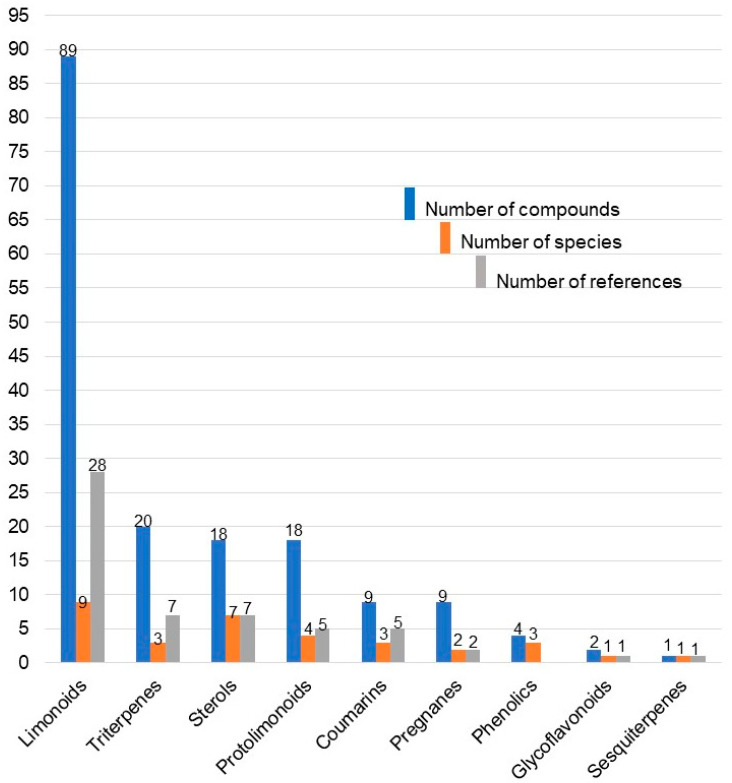
Number of compounds (chemical diversity), classes of compounds, and references for each species of South African Meliaceae.

**Figure 5:**
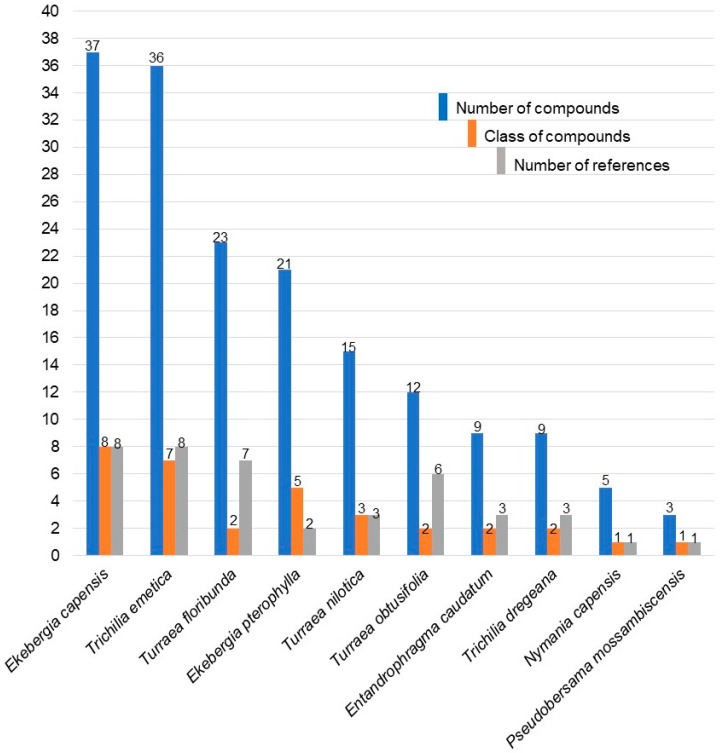
Number of compounds, classes of compounds and references for each species of South African Meliaceae.

The isolated compounds are categorised into limonoids, triterpenes, sterols, protolimonoids, coumarins, pregnanes, phenolics, glycoflavonoids, and sesquiterpenes. The highest number of compounds reported in the studied species are limonoids (89 compounds) followed by triterpenes (20 compounds) (Figure 4). Limonoids were identified in all of the species except *P. mossambicensis* where only sterols have been reported until now (Table 3). Out of the various compounds isolated from South African Meliaceae, the highest number were from *E. capensis* (37) followed by *T. emetica* (36), *T. floribunda* (23), *E. pterophylla* (21), *T. nilotica* (15), *T. obtusifolia* (12), *E. caudatum* (9), *T. dregeana* (9), *N. capensis* (5), and *P.a mossambicensis* (3) (Figure 5). Most of the compounds were isolated from the bark (42%), followed by seed (17%), wood (16%), root (13%), leaf (11%), and twig (1 %) of the plants (Figure 3c).

#### 2.2.2. Structures of Some of the Isolated Compounds

Most of the reported limonoids are summarised into series according to their structure (Table 4 and Figure 6, Figure 7, Figure 8, Figure 9, Figure 10, Figure 11, Figure 12, Figure 13, Figure 14, Figure 15 and Figure 16) or with common structural themes. Floribundin naming is used here for the first time.

## 3. Materials and Methods

The recorded ethnobotanical uses and isolated compounds were based on a search of scopus and science-direct electronic databases, pubMed, reference libraries, conference papers, ethnobotanical books, dissertations, theses, and scientific articles. All of the relevant papers were included in this study except those that were not peer reviewed and those containing species that are not indigenous to South Africa. The ethnomedicinal uses are classified into 17 major categories, based on Moffett’s [150] classification, while the compounds were categorised into nine main chemical classes and the structures drawn using ACD/ChemSketch Freeware (Windows platform).

## 4. Conclusions

The species of South African Meliaceae have been reportedly used for a diversity of purposes from medicinal (including human and animal), to rituals, to functional uses (making of implements, furniture, oils, and dyes). A total of 85 different medicinal uses were recorded, and *T. emetica* is the most frequently used, followed by *E. capensis*. Several compounds have been isolated from South African Meliaceae. A total of 188 compounds belonging to nine classes were recorded from various plant parts. The highest number of compounds belonged to the limonoids class, followed by sterols. There was no record found for the chemistry of *T. streyi* and *T. pulchella*. 

The high chemical diversity of these species may be related to the high diversity of therapeutic uses recorded. South African Meliaceae are mostly used in the treatment of gastro-intestinal ailments followed by gynaecological and obstetrics related ailments. The most common mode of herbal preparation was decoction followed by infusion. The roots followed by bark are mostly commonly used in the preparation of the remedies, whereas most of the compound isolation work focused on the bark followed by seeds – this may be a consequence of logistical difficulty in obtaining roots for chemical study. 

The ethnomedicinal uses recorded in this study are of value for bioprospectors or synthetic chemists looking for chemical scaffolds as precursors to biologically enhanced derivatives. However, the current review also strengthens the call for increased conservation practice, which is due to the fact that root and bark harvesting are destructive. Hence, it may be important to examine the chemistry of plant parts such as leaves and fruits. 

## Figures and Tables

**Figure 6:**
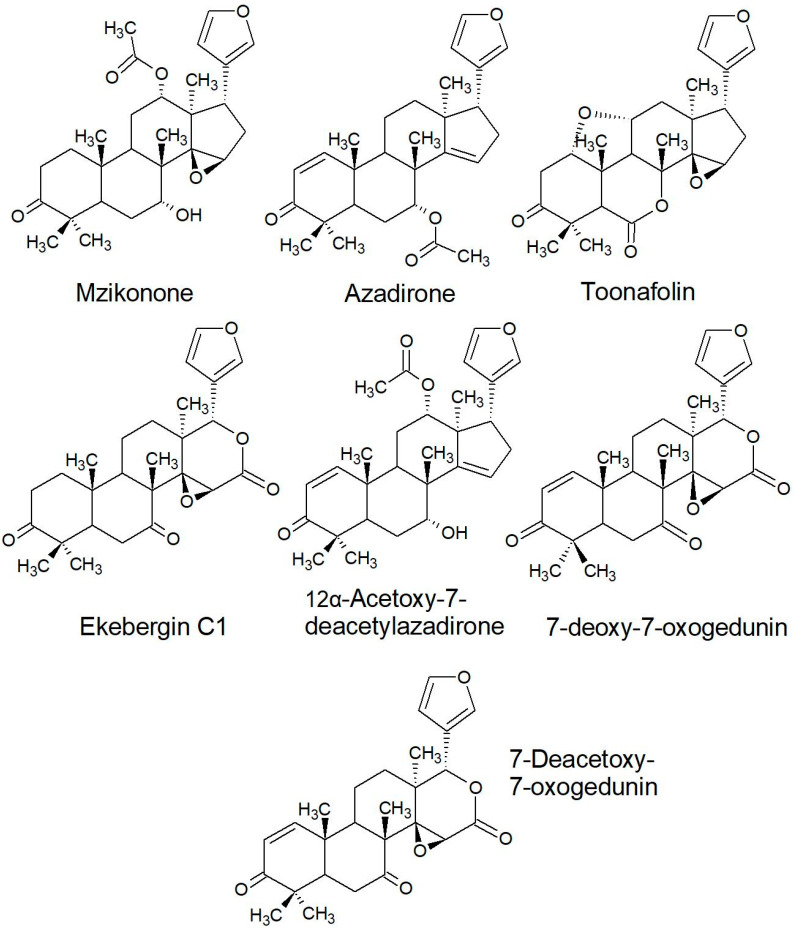
Ketone series of limonoids reported from South African Meliaceae [33,146,147].

**Figure 7:**
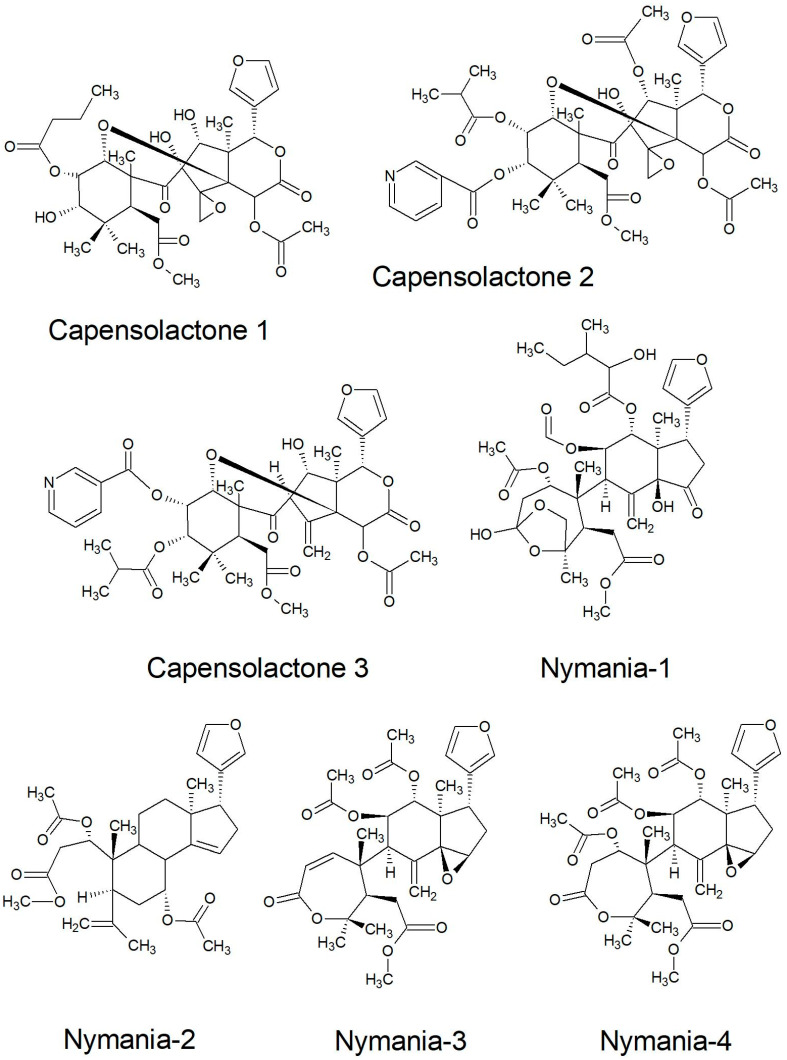
Capensolactones and *Nymania* series of limonoids reported from South African Meliaceae [108,112].

**Figure 8:**
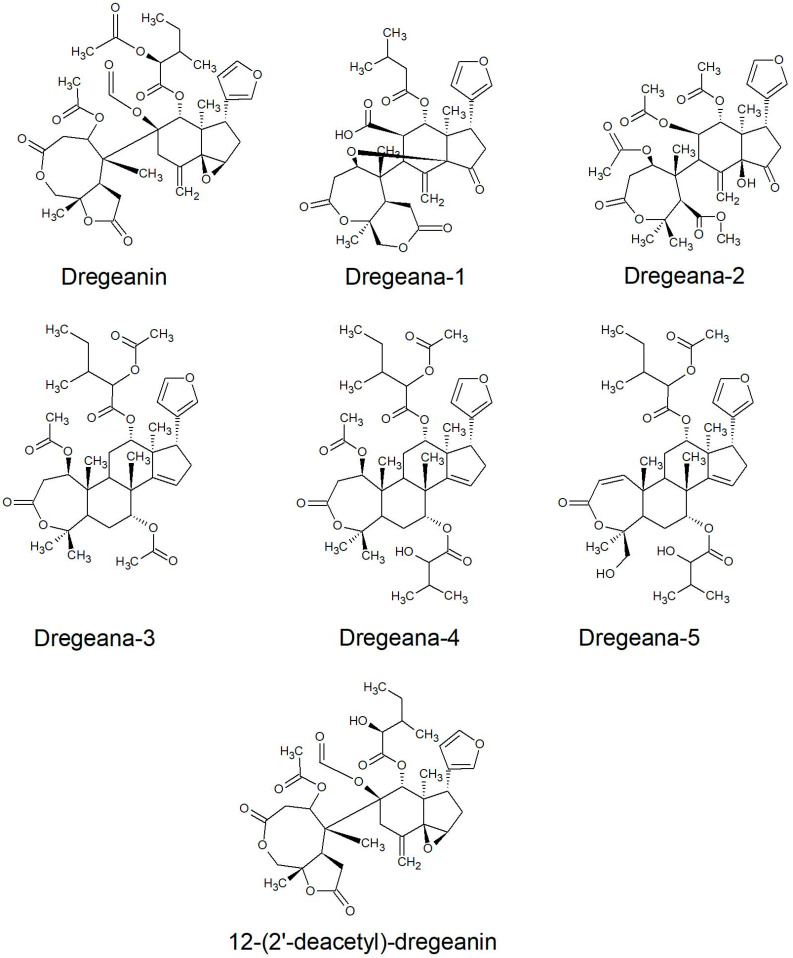
Dregeanin series of limonoids reported from South African Meliaceae [117,136].

**Figure 9:**
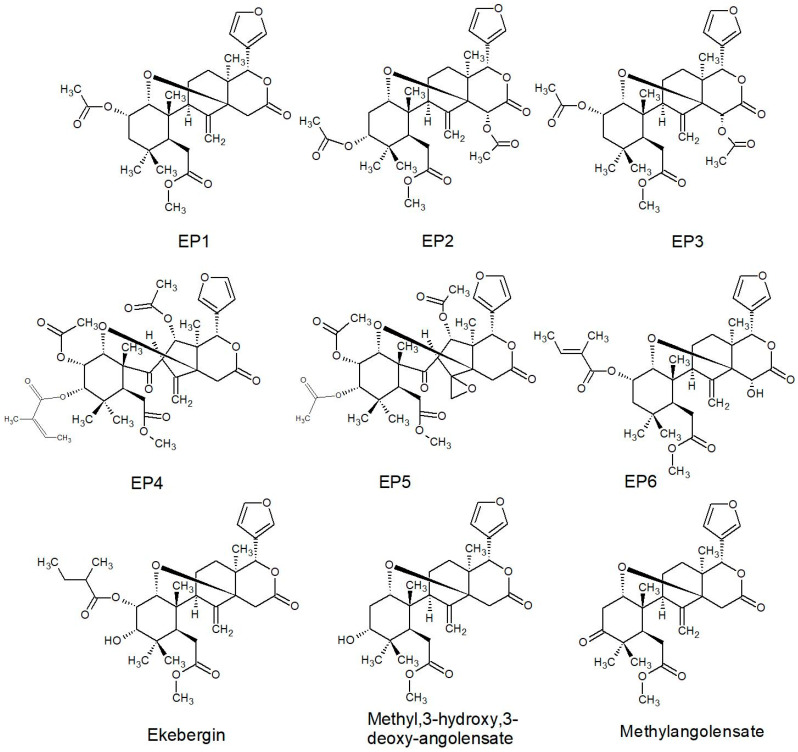
EP series of limonoids reported from South African Meliaceae [33,108,110,111].

**Figure 10:**
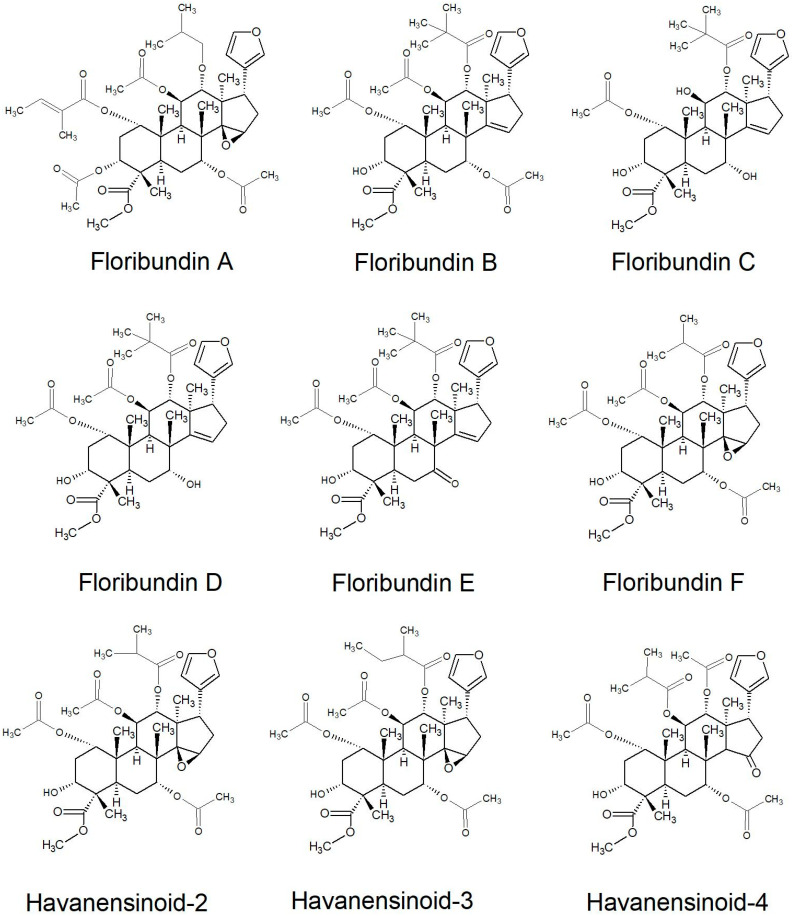
Floribundin and Havanen series of limonoids reported from South African Meliaceae [121,141,142,143].

**Figure 11:**
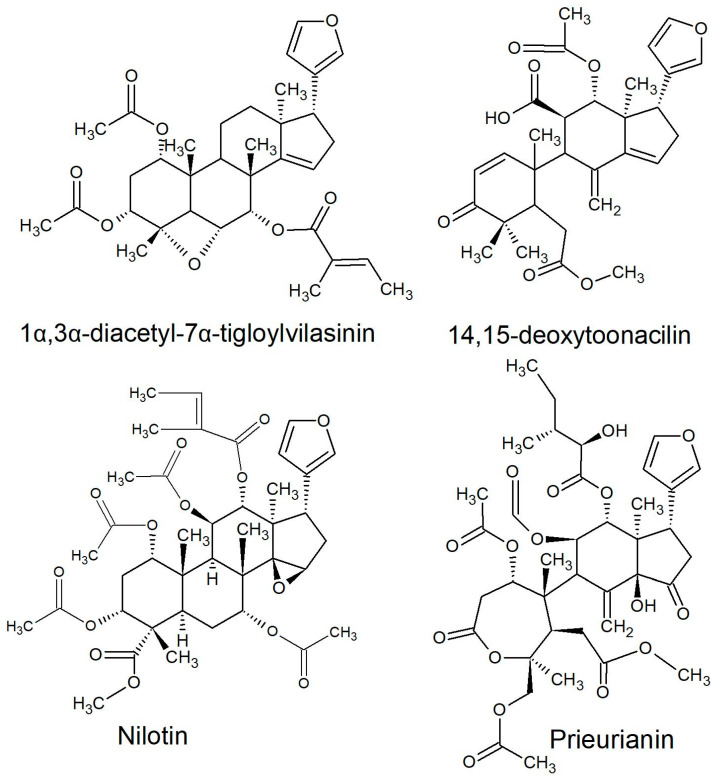
Orphan series of limonoids reported from South African Meliaceae [108,119,121,147].

**Figure 12:**
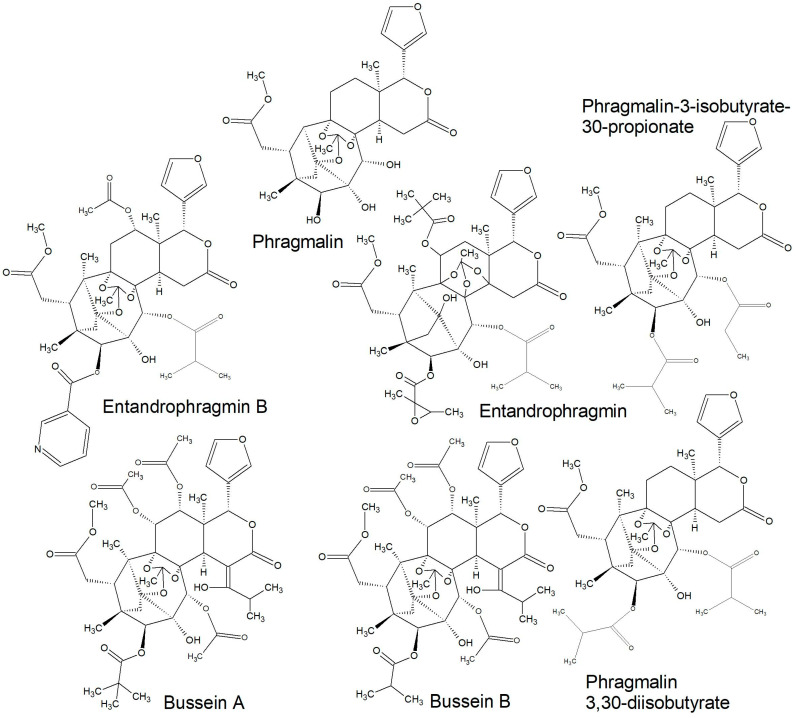
Phragmalin, phragmin, and bussein series of limonoids reported from South African Meliaceae [113,116].

**Figure 13:**
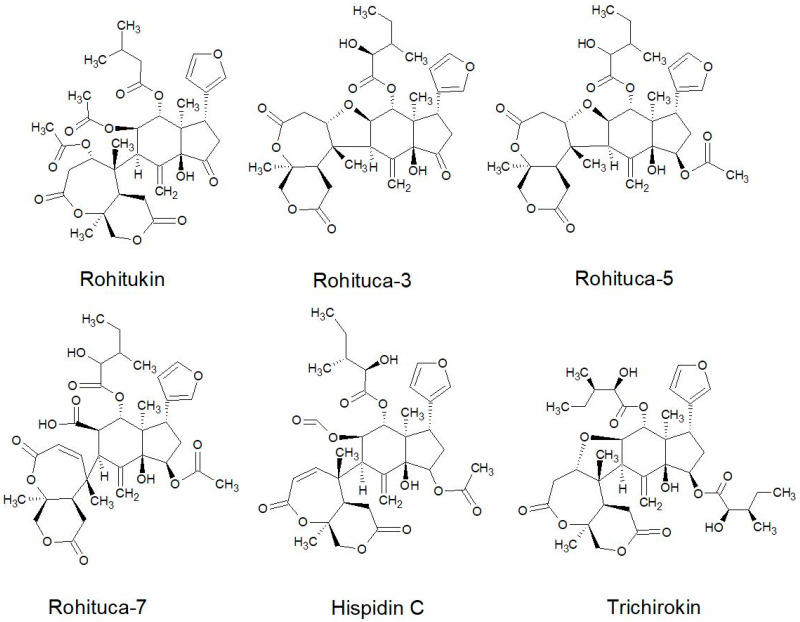
Rohituka series of limonoids reported from South African Meliaceae [24,117,118].

**Figure 14:**
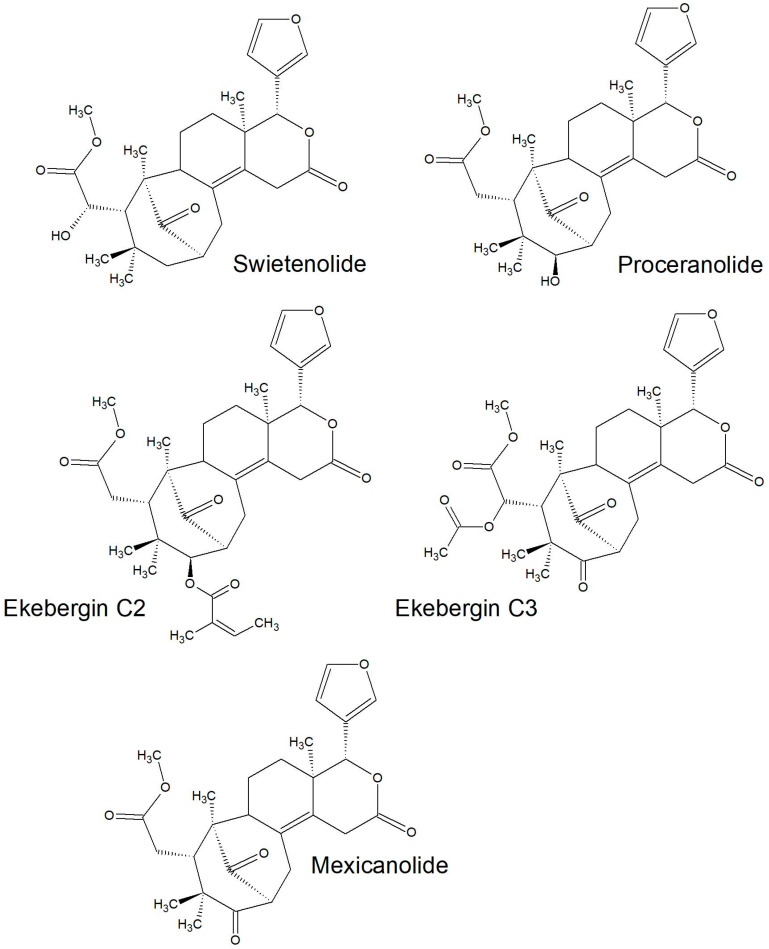
Swietenolide and Ekebergin series of limonoids reported from South African Meliaceae [31,33].

**Figure 15:**
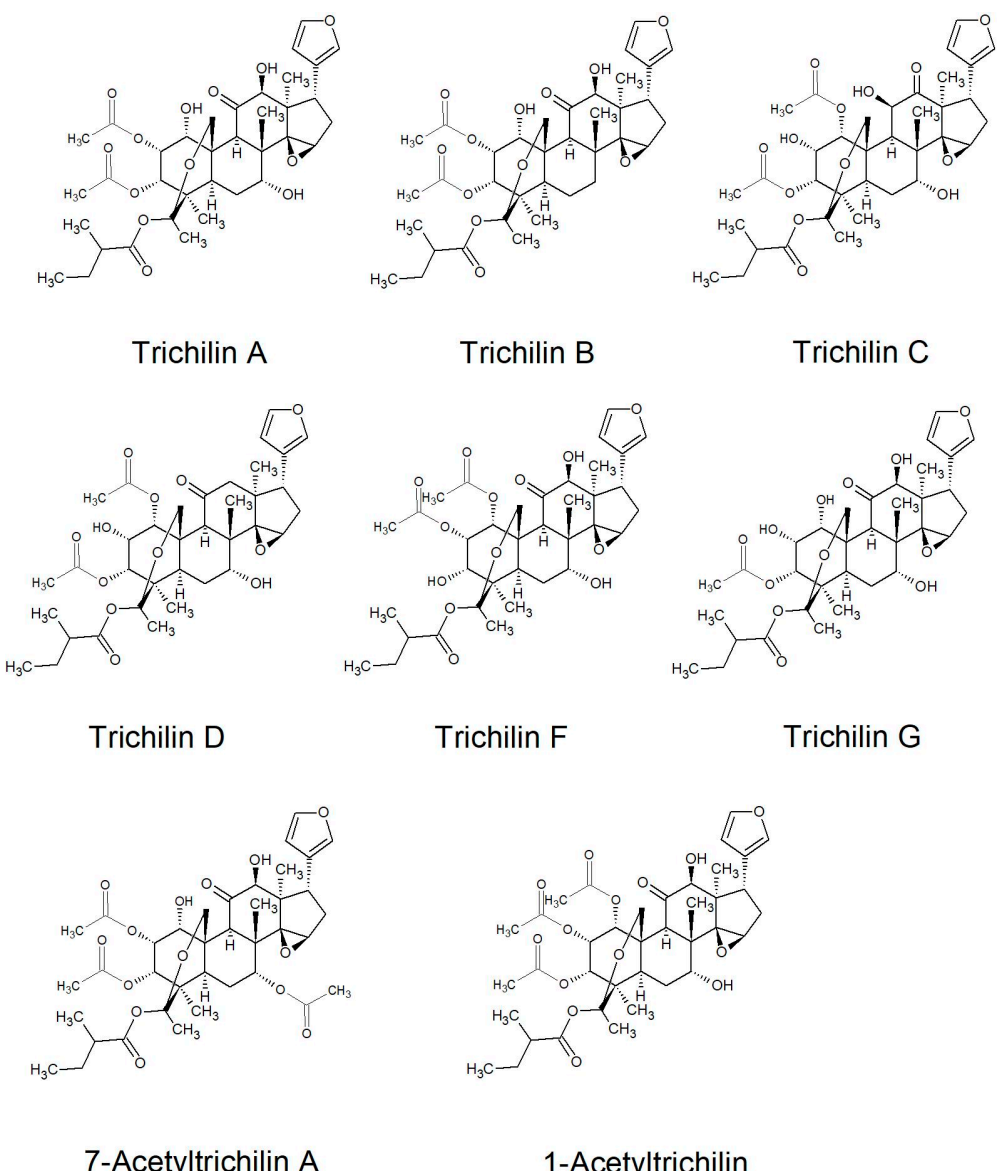
Trichilin series of limonoids reported from South African Meliaceae [137,138,139].

**Figure 16:**
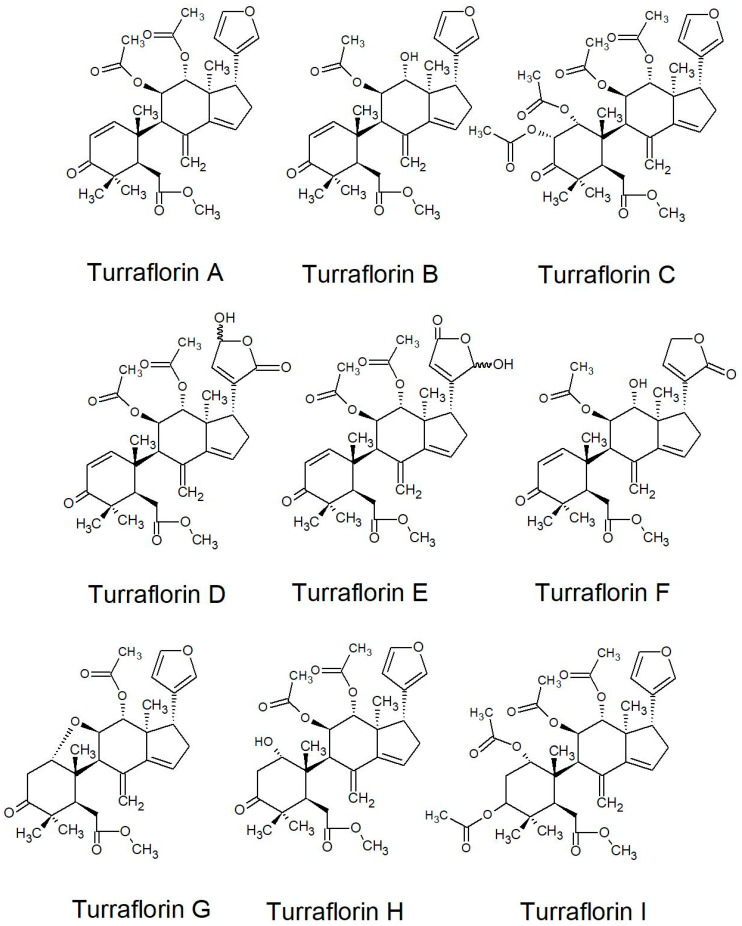
Turraflorin series of limonoids reported from South African Meliaceae [112,144].

**Table 2:** The functional uses of South African Meliaceae.

Taxa	Traditional Use			References
	Functional Use	Part Use	Method of Use	
*Ekebergia capensis*	Tanning, furniture, brush, broom heads and handle, beams, planks, wagon, ship and boat building, light construction, poles and tool handles, light flooring, joinery, interior trim, vehicle bodies, sporting goods, toys, novelties	Wood	Wood is used in furniture industry	[12,40]
	Firewood and charcoal production	Wood	Wood is used in cooking	[12,40]
	Animal feed	Fruit and leaf	Birds feed on fleshy parts of the fruit, while the leaf is used as a fodder	[12,40]
	Shades and wind break	Whole plant	It serves as an ornamental tree planted in gardens and roadsides for shades, as well as for wind break and soil conservation	[40,43]
	Edible caterpillar	Whole plant	Caterpillars are gathered from the plant and prepared as food	[43,101]
*Ekebergia pterophylla*	Garden tree	Whole plant	Whole plant is used as an ornamental garden tree as well as a bonsai	[76]
*Entandrophragma caudatum*	Furniture, cabinet making, carving canoes	Wood	Wood is light and durable, hence high demand by the furniture industry	[8]
	Tanning	Wood	Wood sap is used for tanning	[43]
	Toy	Fruit	Fruit pericarp is used to make ‘zwihwilili’ with which children like to play	[43,101]
	Shade	Whole plant	Whole plant is favoured for shade	[43]
	Animal feed	Seed	Seed is eaten by antelope	[75]
*Nymania capensis*	Forage	Leaf	Source of forage for goats	[89]
				
*Pseudobersa mamossam bicensis*	Buildings and charcoal	Wood	Wood is used in making poles in local house buildings, as well as firewood and making charcoal	[102]
*Trichilia dregeana*	Furniture and carving	Wood	Wood is used for carving, repair of ships, and for making household furniture	[8,55,75]
	Craftwork	Wood	NR	[101]
	Food condiments	Fruit	Fruit content is cooked with vegetables, fruit pulp is eaten as sour milk, while the oil made from the fruit pulp is used in cooking vegetables and other relishes	[43,65]
	Polish	Fruit and seed	Oil made from seed and fruit pulp is used to polish women’s clothes made from leather, furniture, and other household implements made from wood	[43]
	Soap and cosmetics	Seed	Oil from seeds is used to make soap, cosmetics, and candles	[43,75,103]
	Forage or fertilizer	Seed	Residue from seeds after oil extraction is used as a fertilizer or animal feed	[65]
		Fruit	Fruits are eaten by birds and bats	[75]
	Drink	Seed aril	Seed aril is pounded and made into a sauce or sweet drink	[65]
	Shade	Whole plant	Whole plant is used to create shady avenue and as an ornamental tree	[65,75]
	Fishing	Seed	Bright-coloured seed is used as bait for fishing	[65]
*Trichilia emetica*	Shade	Whole plant	Whole plant is used for shade	[43,75]
	Soap and cosmetics	Seed	Oil from seed is used to make soap, body ointment, and candle	[8,71,76,103,104]
	Fertilizer	Seed	Residue from seed after oil extraction is used as a fertilizer	[71]
	Kola	Seed aril	Seed aril is eaten as a substitute for kola	[71]
	Forage	Leaf	Leaf is eaten by cattle and goats	[71]
		Fruit	Fruit is eaten by baboons, antelopes, and monkeys	[75]
		Seed	Seed is eaten by birds	[75,76]
	Carvings	Wood	Wood is used in carving furniture, household implements, musical instruments, canoes, and as chew-stick	[8,71,75,76,105]
	Dyeing	Bark	Pinkish or light red-brown dye is obtained from the beaten boiled bark	[4,71,75]
	Cooking	Seed aril	Seed aril is soaked and cooked together with squash or sweet potatoes	[8,75,101]
	Multivitamin	Seed	Juice is made from the seeds and other edible plants to control malnutrition	[106]
	Beverage	Fruit	NR	[101]
*Turraea floribunda*	Traps	Wood	Wood is used for making traps	[107]
	Ornamental	Whole plant	Tree is used as an ornamental plant in humid, frost-free subtropical and tropical gardens, and as a greenhouse plant in temperate countries	[76,82]
*Turraea nilotica*	Handicrafts	Stem/branches	Stems/branches are used for handicrafts and domestics purposes	[107]
	Firewood	Branches	Branches are sorted for firewood	[107]
*Turraea obtusifolia*	Ornamental	Whole plant	Plant is used as an ornamental container plant in landscape designs, as well as an attractive garden plant	
	Insect repellent	Leaf	Insect repellent	[87,101]

**Table 4:** Reported limonoids according to series.

Series	Limonoids
Ketone and Azadirone	Mzikonone; Azadirone; Toonafolin; Ekebergin C1; 12α-Acetoxy-7-deacetylazadirone; 7-deoxy-7-oxogedunin; 7-deacetoxy-7-oxogedunin
Capensolactones and *Nymania*	Capensolactone 1-3; *Nymania* 1-4
Dregeanin	Dregeanin; *Dregeana* 1-5; 12-(2′-deacetyl)-dregeanin
EP *	EP1-6; Ekebergin; Methyl,3-hydroxy,3-deoxy-angolensate; Methlyangolensate
Floribundin ** and Havanen	Floribundin A-F; Havanensinoid 2-4
Orphan	1α,3α-diacetyl-7α-tigloylvilasinin; 14,15-deoxytoonacilin; Nilotin; Prieurianin
Phragmalin and Phragmin	Entandrophragmin B; Phragmalin 3-isobutyrate-30-propionate; Entandrophragmin; Phragmalin 3,30-diisobutyrate; Phgramalin; Bussein A and B
Rohituka	Rohituca 3, 5 and 7; Hispidin C; Trichirokin
Swietenolide/Ekebergin	Swietenolide; Proceranolide; Ekebergin C2-C3; Mexicanolide
Trichilin	Trichilin A-G; 7-Acetyltrichilin A; 1-Acetyltrichilin
Turraflorin	Turraflorin A-I

* EP is an abbreviation of *Ekebergia pterophylla* (Taylor and Taylor 1984; Kehrli et al., 1990; Mulholland et al., 1998; Murata et al., 2008) ** Floribundin A–F was named by the authors the since the authors did not provide a shorter name.

## Data Availability

Not applicable.
